# Multiple-Layer Visibility Propagation-Based Synthetic Aperture Imaging through Occlusion

**DOI:** 10.3390/s150818965

**Published:** 2015-08-04

**Authors:** Tao Yang, Jing Li, Jingyi Yu, Yanning Zhang, Wenguang Ma, Xiaomin Tong, Rui Yu, Lingyan Ran

**Affiliations:** 1 ShaanXi Provincial Key Lab of Speech and Image Information Processing, School of Computer Science, Northwestern Polytechnical University, Xi’an 710129, China; E-Mails: ynzhangnwpu@gmail.com (Y.Z.); tongxiaominnwpu@gmail.com (X.T.); ranlingyannwpu@gmail.com (L.R.); 2 School of Telecommunications Engineering, Xidian University, Xi’an 710126, China; E-Mail: jinglixd@mail.xidian.edu.cn; 3 Department of Computer and Information Science, University of Delaware, Newark, DE 19716, USA; E-Mails: yu@eecis.udel.edu (J.Y.); 18392649664@163.com (W.M.); 4 Department of Computer Science, University College London, London, WC1E 6BT, UK; E-Mail: r.yu@cs.ucl.ac.uk

**Keywords:** camera array, multiple-layer visibility propagation, occluded object imaging, all-in-focus synthetic aperture imaging

## Abstract

Heavy occlusions in cluttered scenes impose significant challenges to many computer vision applications. Recent light field imaging systems provide new see-through capabilities through synthetic aperture imaging (SAI) to overcome the occlusion problem. Existing synthetic aperture imaging methods, however, emulate focusing at a specific depth layer, but are incapable of producing an all-in-focus see-through image. Alternative in-painting algorithms can generate visually-plausible results, but cannot guarantee the correctness of the results. In this paper, we present a novel depth-free all-in-focus SAI technique based on light field visibility analysis. Specifically, we partition the scene into multiple visibility layers to directly deal with layer-wise occlusion and apply an optimization framework to propagate the visibility information between multiple layers. On each layer, visibility and optimal focus depth estimation is formulated as a multiple-label energy minimization problem. The layer-wise energy integrates all of the visibility masks from its previous layers, multi-view intensity consistency and depth smoothness constraint together. We compare our method with state-of-the-art solutions, and extensive experimental results demonstrate the effectiveness and superiority of our approach.

## 1. Introduction

The capability of seeing through occlusions in heavily-cluttered scenes is beneficial to many practical computer vision application fields, ranging from hidden object imaging to detection, tracking and recognition in surveillance. Since traditional imaging methods use a simple camera to acquire the 2D projection of the 3D world from a single viewpoint, they are unable to directly resolve the occlusion problem.

**Figure 1:**
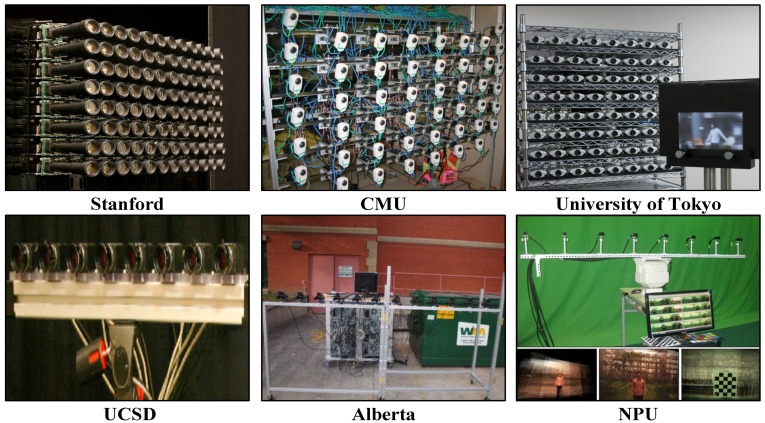
Examples of camera array synthetic aperture imaging sensors. NPU, Northwestern Polytechnical University.

A fundamental solution to the problem is to exploit new imaging procedures. For example, emerging computational photography techniques based on generalized optics provide plausible solutions to capture additional visual information. Many different camera arrays have been built over the past few years (as shown in Figure 1), and the camera array synthetic aperture imaging or SAI [1,2,3,4,5,6,7,8,9,10,11,12,13,14,15,16,17] provides a unique capability of seeing through occlusions. SAI warps and integrates the multiple view images to simulate a virtual camera with an ultra-large convex lens, and it can focus on different frontal-parallel [1] or oblique [2] planes with a narrow depth of field. As a result, objects laying on the virtual focus plane, even if being occluded in reality, would be clearly imaged.

In practice, however, getting a good imaging result is still challenging. For one thing, we really can’t find the focus plane of an object for all of those cameras without sufficient visibility analysis. Actually, the indiscriminate averaging of the projection of the foreground occluder often significantly blurs the images of focused occluded objects. Additionally, objects laying on different focus planes in the scene always fail to be present in one single synthetic image.

### 1.1. Our Approach

**Figure 2:**
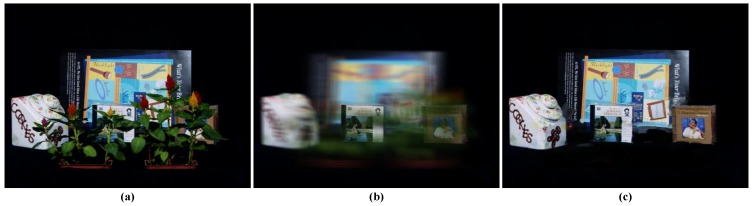
Comparison results of occluded object synthetic aperture imaging methods. (**a**) One camera view in the Stanford camera array; (**b**) Result of traditional synthetic image focused on the CD case; (**c**) Result of occluded object all-in-focus image by our method.

To overcome the weakness above, we present a novel synthetic aperture imaging algorithm, which aims at generating a depth-free all-in-focus synthetic image (as shown in Figure 2c). Here, the all-in-focus image refers to a synthetic one that contains not only objects laying on the virtual focus plane, but also those that are not. Depth-free means that, given a certain depth, the algorithm can see through occluders and generate a clear all-in-focus image of the scene contents behind it.

Different from in-painting algorithms [18,19], which can generate visually-plausible results, but not guarantee the correctness of the result, our technique is based on the light field visibility analysis. For every 3D point, we trace all rays passing through it back to the camera array and then construct a visibility layer in which the 3D point is visible in all active cameras. To recover the all-in-focus image behind a specific depth layer, we partition the scene into multiple visibility layers to directly deal with layer-wise occlusion and apply an optimization framework to propagate the visibility information between multiple layers. On each layer, visibility and optimal focus depth estimation is formulated as a multiple label energy minimization problem. The energy integrates the visibility mask from previous layers, multi-view intensity consistency and the depth smoothness constraint. We compare our method with state-of-the-art solutions on publicly-available Stanford [20] and UCSD (University of California, San Diego) [21] light field datasets and three datasets captured by ourselves with multiple occluders. As illustrated in Figure 2, conventional synthetic aperture imaging algorithms only focus on a particular depth and result in poor quality (see Figure 2b), while our approach, which creatively segments the scene into multiple visibility layers and uses an optimization framework to propagate the visibility information between multiple layers, can produce the all-in-focus image even under occlusion (see Figure 2c). Extensive experimental results with qualitative and quantitative analysis further demonstrate the performance.

### 1.2. Related Work

Tremendous efforts have been made on developing light field imaging systems and post-processing algorithms. On the hardware front, light field camera arrays with different numbers of cameras, resolution and effective aperture size have been built (as shown in Figure 1), e.g., Stanford [3], CMU [4], UCSD [5], Alberta [6], Delaware [7], Northwestern Polytechnical University (NPU) [8,16], PiCam [15], Tokyo [17], *etc.*, and the camera array synthetic aperture imaging technique has been proven to be a powerful way to see objects through occlusion. Similar camera array techniques have been adopted in producing movie special effects. For instance, in the 1999 movie The Matrix, a 1D camera array is used to create an impressive bullet dodging scene that freezes time, but changes viewpoint towards the character.

On the algorithm front, one of the most important techniques is synthetic aperture imaging (SAI). By integrating appropriate rays in the camera array, SAI can generate a view that would be captured by a virtual camera having a large aperture. In addition, through shearing or warping the camera array images before performing this integration, SAI can focus on different planes in the scene. For example, the Stanford LF (Light Field) camera array by Levoy *et al.* [3] consists of 128 Firewire cameras, and for the first time, aligns multiple cameras to a focus plane to approximate a camera with a very large aperture. The constructed synthetic aperture image has a shallow depth of field, so that objects off the focus plane disappear due to significant blur. This unique characteristic makes synthetic aperture imaging a powerful tool for occluded object imaging.

Taking advantage of the geometry constraints of the dense camera array, Vaish *et al.* [11] present a convenient plane + parallax method for synthetic aperture imaging. A downside of their work, however, is that all rays from the camera array are directly integrated without further analysis. Thus, the clarity and contrast of their imaging result would be reduced by rays from the foreground occluders.

Visibility analysis through occlusion is a difficult, but promising way to improve the occluded object imaging quality, and many algorithms have been developed in this way. Vaish *et al.* [12] study four cost functions, including color medians, entropy, focus and stereo, for reconstructing an occluded surface using synthetic apertures. Their method achieves encouraging results under slight occlusion; however, the cost functions may fail under severe occlusion. Joshi *et al.* [10] propose a natural video matting algorithm using a camera array. Their method uses high frequencies present in natural scenes to compute mattes by creating a synthetic aperture image that is focused on the foreground object. Their result is inspiring, and it has the potential to be used for visibility analysis. However, this algorithm may fail in the case of a textureless background and cannot deal with occluded object matting. Pei *et al.* [13] propose a background subtraction method for segmenting and removing foreground occluders before synthetic aperture imaging. Their result is encouraging in a simple static background; however, since this approach is built on background subtraction, it cannot handle static occluders. In addition, their performance is very sensitive to a cluttered background and may fail under crowded scenes.

The most relevant method to ours would be the work of Pei *et al.* [6], which solves the foreground segmentation problem through binary labeling via graph cuts. Instead of labeling the visibility and focusing depth, they label whether a point is in focus at a particular depth and aggregate these focus labels in a given depth range to get a visibility mask for occluded object imaging. Although the result is encouraging, this method can only deal with front occluder (whose depth range needs to be provided as a prior) labeling problem and may fail if the occluder has severe self-occlusion or there are multiple occluded objects due to a lack of visibility propagation. In addition, the result of the method in [6] can only focus on a particular depth of the scene instead of all-in-focus imaging, and the performance will be decreased in a textureless background.

A graph cut [22,23,24,25] is one of the most popular methods to solve the Markov random field (MRF) energy minimization problem due to its efficiency. In this paper, we formulate the entire scene synthetic aperture imaging as a multi-layer multi-labeling problem, which can be solved via graph cuts. Different from most works using graph cuts for 3D scene reconstruction, in our algorithm, we creatively apply the graph cuts in solving the visibility property and optimal focus depth labeling energy minimization problems. In order to solve the visibility property labeling energy minimization problem, we carefully design a key energy function with a different data term, which can measure the cost of assigning a visibility label to a pixel, and a different smoothness term, which can encourage neighboring pixels to belong to the same visibility label. Additionally, in order to solve the optimal focus depth labeling energy minimization problem, we carefully design another key energy function whose data term can measure the cost of assigning a depth layer to a pixel and whose smoothness term can courage neighboring pixels to share the same label.

The organization of this paper is as follows. Section 2 presents the visibility layer propagation-based imaging model. Section 3 details the visibility optimization algorithm. Section 4 details the visibility optimization algorithm. Section 5 describes the dataset, implementation details and the experimental results. We conclude the paper and point out the future work in Section 6.

## 2. Visibility Layer Propagation-Based Imaging Model

**Figure 3:**
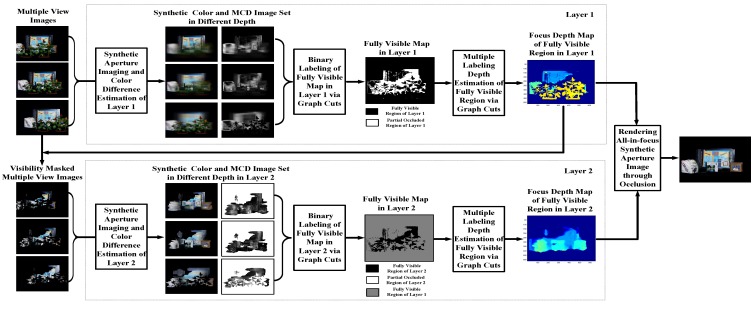
Framework of our active synthetic aperture imaging algorithm. MCD, maximal color difference.

In this section, we will introduce our multiple-layer propagation-based synthetic aperture imaging method. The framework of our method is an iterated process (as shown in Figure 3). The scene is divided into different visibility layers. For each layer, we have two steps, visibility labeling and depth estimation, which will be discussed in Section 3 and Section 4, respectively.

Instead of segmenting the observed scene into various depth layers, our approach segments the entire scene into multiple visibility layers, for example the Stanford Light Field dataset shown in Figure 3 is divided into two visibility layers. The visibility layer is defined on each layer as all of the rays that are not occluded in any cameras and computed by energy minimization. Points on each visibility layer do not necessarily need to correspond to the same object or surface. By modeling the scene as multiple visibility layers and propagating visibility information through layers, we can obtain the focusing depth and corresponded cameras for all of the objects in the scene, including the occlusion object and occluded objects. Therefore, each visibility layer consists of pixels that are visible in all active cameras. The word active refers to the fact that the pixel position of the camera has not been labeled as visible by the previous visibility layers, e.g., not labeled as visible by Layer 1 in Figure 3. Extraction of each visibility layer is based on the information of previous visibility layers. More precisely, according to the occlusion mask information of previous layers, we firstly obtain the current visibility layer, then estimate the depth map of this layer and, finally, update the occlusion mask. For better understanding of the proposed method, we provide an example workflow with the Stanford Light Field data in Figure 3.

There are mainly two reasons why we introduce the concept of the visibility layer. First, by taking advantage of the introduced visibility layer, the occlusion problem can be tackled more directly. The visibility information is propagated from layer to layer, and in each layer, the occlusion mask needs to be updated only once. Second, segmenting the scene into visibility layers instead of depth layers is more beneficial, as neighboring pixels in the same layer tend to belong to the same object, and the depth smoothness constraint can be enforced when estimating the depth map.

Let *L* denote the number of visibility layers in the scene. For each layer, we need to find a labeling function f:Ω→ℒ, where Ω refers to the set of all unmasked pixels in all images and $\text{ℒ}=\{0,d_{1},d_{2},\ldots ,d_{m}\}$ denotes the set of possible labels of these pixels. $d_{i}\left(i=1,2,\ldots ,m\right)>0$ represents the depth range of our scene. For a pixel x, if $f\left(\text{x}\right)>0$, then x is fully visible in all active camera views. Otherwise, if $f\left(\text{x}\right)=0$, then x is partially occluded.

Considering the labeling redundancy of camera array (the labels in different cameras are highly related), the problem can be further simplified. Instead of labeling all of the unmasked pixels of all of the cameras, we label all of the pixels of the reference camera equivalently (not only the unmasked pixels, as a masked pixel of the reference camera may still be fully visible in all of the other active cameras). This means that if there are *N* cameras in the camera array, we only label all of the pixels of the reference camera view instead of labeling all of the unmasked pixels of all cameras. Specifically, instead of finding the above labeling function, we seek a more succinct labeling, $g:I_{ref}\to \text{ℒ}$, where $I_{ref}$ refers to the whole image area of the reference camera. In our implementation, the visibility and depth map is calculated first on the reference image, then the visibility and depth maps of all of the other cameras are derived based on the calibration information of the camera array (as shown in Figure 3, the visibility masked multiple view images).

Therefore, for each layer *ℓ*, the problem of estimating fully-visible pixels and corresponding depths can be formulated as the following energy minimization problem:

(1) $$\begin{array}{c} E\left(g;V_{1},V_{2},\ldots ,V_{\ell -1}\right)=E_{d}\left(g\right)+E_{s}\left(g\right) \end{array}$$

where the data term $E_{d}$ is a data penalty function, which can measure the cost of assigning the label *g* to the pixel, and the smooth term $E_{s}$ is a regularizer that encourages neighboring pixels to share the same label, while the visibility information $V_{k}\left(k=1,2,\ldots ,\ell -1\right)$ from previous layers is used to encode and block the occluded rays. In our algorithm, the estimation of visibility information is coupled to the depth value, and it is difficult to minimize energy Function (1) directly, so we let the label *g* represent two kinds of labeling information, including visibility labeling information and depth labeling information. Additionally, if we want to get the visible map of each visibility layer, we can make $g=V_{\ell }$, so Equation (1) can be transformed into Equation (2) and can be solved in Section 3 through the carefully-designed data term and smoothness term. Additionally, if we want to get all depth maps of each visibility layer, we can make $g=D_{\ell }$, so Equation (1) can be transformed into Equation (9) and can be solved in Section 4 with the well-designed data term and smoothness term.

As the estimation of visibility information is coupled to depth value and can only be obtained by analyzing synthetic images of different depths of focus, it is difficult to minimize energy Function (1) directly. In this paper, we solve for $g=\{V_{\ell },D_{\ell }\}$ by the following two optimization modules: (1) optimize the visible map $V_{\ell }$ in the reference camera (as shown in Figure 3, the fully-visible map in Layers 1 and 2); (2) calculate the depth map $D_{\ell }$ of visible pixels (as shown in Figure 3, the focus depth map of the fully-visible region in Layers 1 and 2).

In the first module, in order to obtain the fully-visible map, we formulate this problem as a binary energy minimization problem, which can be optimized by graph cuts [22]. The energy function used in the first module is composed of an energy function with a unary data term, which represents the cost of assigning a visibility label to a pixel, and a pairwise smoothness term, which accounts for the smoothness prior of the visibility layer. More details about the first module will be discussed in the Section 3.

In the second optimization module, estimation of the optimal focus depth for pixels in each visible layer is formulated as a multiple-label energy minimization problem and is also solved via graph cuts [22]. The energy function is composed by a unary data term, which indicates the cost of assigning a depth label to a pixel, and a pairwise smoothness term, which accounts for the smoothness constraint of the depth map. More details about the second optimization module will be discussed in the Section 4.

## 3. Multiple-Layer Visibility Optimization

Since our method propagates the binary visible map between multiple layers, for a certain layer ℓ∈{1,2,…,L}, occluders in front of this layer have been labeled and can be easily removed in the images of all cameras. To make the notation uncluttered, we do not write previous visibility layers $V_{k}\left(k=1,2,\ldots ,\ell -1\right)$ explicitly unless necessary. As a result, the visibility energy function can be written as follows:

(2) $$E\left(V_{\ell }\right)=E_{d}\left(V_{\ell }\right)+E_{s}\left(V_{\ell }\right)$$

Data term: If a pixel is fully visible in the current layer, it should be in focus for some depth value, and at the same time, corresponding pixels that form the synthetic aperture image should be related by the same point of an object (except those occluded by previous layers). If a scene point is in focus, its corresponding pixel in the synthetic aperture image will have good clarity and contrast, which can be measured by state-of-the-art focusing metrics. In addition, the corresponding pixels that form the synthetic aperture image should have a similar intensity value, which can be measured by various intensity constance metrics. In this paper, focusing metrics and intensity constance metrics are all referred to as focusing metrics. We define the cost of labeling a pixel as fully visible based on its corresponding curve of focusing metrics in synthetic images of different depths of focus.

**Figure 4:**
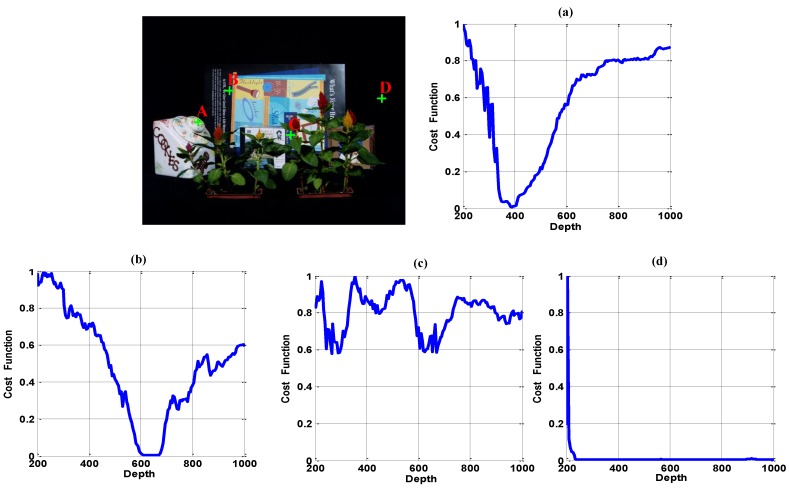
Typical focusing curve of different kinds of points. (**a**) Point A: Fully-visible texture region; (**b**) Point B: Fully-visible region with pure color; (**c**) Point C: Partially-occluded region or free point; (**d**) Point D: Textureless region.

The ideal curve of a fully-visible pixel (Figure 4, Point A) should satisfy the following two constraints: (1) it is unimodal throughout the focus depth scope; and (2) the curve reaches a global minimal, if and only if all visible rays intersect at the same point on an object in the scene. In contrast, a partially-occluded pixel or a free point without focus should always have a large value through the entire focus depth scope (Figure 4, Point C). That is because these points are only visible in some of the cameras, thus for unfocused depth and even for focused depth, the cost of those points is high. A textureless object pixel should have a small value in a small range of depths around the focusing depth (Figure 4, Point B), while a textureless background pixel should have a small value over a broad focus range due to its similarity with the neighborhood pixels (Figure 4, Point D). Besides, Figure 4, Point D gives a sharp peak near the origin. That is caused by the position of focusing depth plane: when it is too close to the camera, the out-of-focus problem results in an unexpected value.

Reasonably, we cannot estimate the depth of the textureless background pixels. Thus, according to the width of the low value depth range, we remove the textureless background region before our binary visibility optimization.

**Figure 5:**
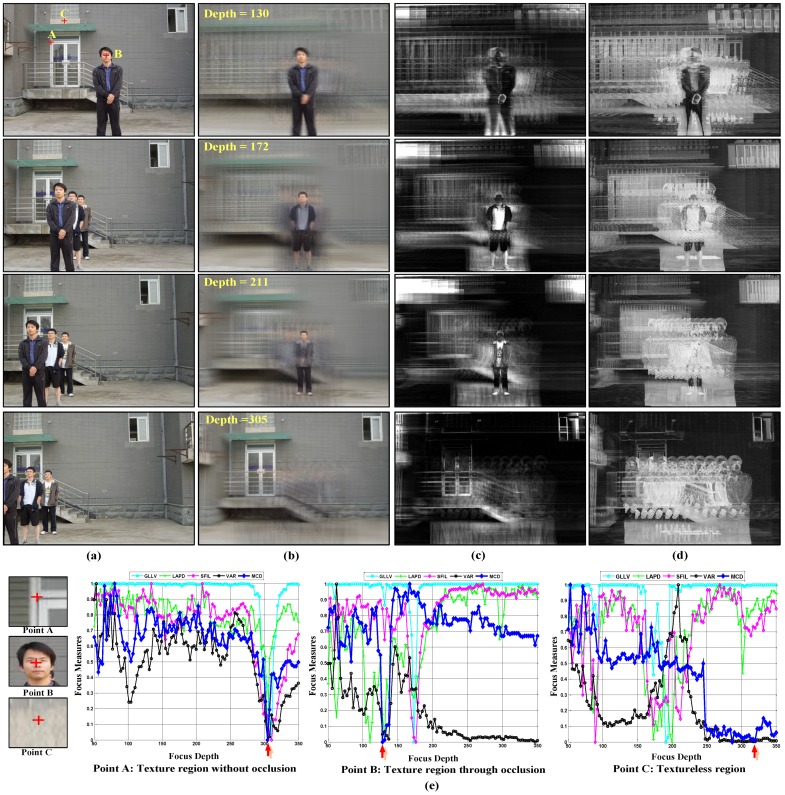
Comparison results of focus measures for different kinds of points; the manually-labeled ground truth focus depth is marked with the red arrow. (**a**) Multiple view images; (**b**)Synthetic Aperture Image; (**c**) Variance Image; (**d**) Our MCD Image; (**e**) Comparison results of different focus measures, variance and maximal color difference metric.

Based on the above analysis, we have compared different kinds of metrics to obtain the desired ideal curve. Part of the comparison result is shown in Figure 5. Figure 5a gives the input images of different cameras, while Figure 5b–d displays the synthetic aperture imaging result, variance image and maximal color difference (MCD) image in different depths. Comparing Figure 5c,d, we can see that the MCD image could describe the minimal color difference more accurately than the variance image. Thus, the MCD measurement is more suitable for visibility analysis.

Figure 5e shows the corresponding curves of Points A–C marked in Figure 5a. The focus measures evaluated include DCTenergy ratio (DCTR) [26], diagonal Laplacian (LAPD) [27], steerable filters (SFIL) [28], variance and MCD. For the first three focus measures, we compute the focus metric using a 5 × 5 pixel block on one hundred sampled focus planes. All of the results are normalized and mapped to [0 1], where a low value represents good focus. The result indicates that for a point in a textured region without occlusion, all focus measures can successfully find the focus point (Point A in Figure 5e). However, when the textured point is occluded in some cameras (Point B in Figure 5e), the curves of DCTR, LAPD, SFIL and variance measures are multimodal with multiple local minima. In contrast, the MCD metric is more stable and more insensitive to occlusion. In the low texture region (Figure 5e, Point C), the first three measures contain many noises. In contrast, both the variance and MCD measure reach the global minimum around the ground truth. In addition, the MCD curve is sharper than the variance and closer to the ideal curve.

Based on the analysis above, we select the MCD measure to define the data cost $E_{d}\left(V_{\ell }\right)$ for each pixel x in the reference camera:

(3) $$E_{d}\left(V_{\ell }\right)=\sum_{\text{x}\in I_{ref}}\left(V_{\ell }\left(\text{x}\right)-\left(1-\min_{d\in D}\left({MCD}^{d}\left(\text{x}\right)\right)\right)\right)$$

where $\text{𝒟}=\{d_{1},d_{2},\ldots ,d_{m}\}$ is the depth range of the scene, ${MCD}^{d}\left(\text{x}\right)\left(d\in \text{𝒟}\right)$ is the MCD focus measure value of the pixel x in depth *d*:

(4) $${MCD}^{d}\left(\text{x}\right)=\max_{\forall i\neq j}\left(|I_{i}^{d}\left(\text{x}\right)-I_{j}^{d}\left(\text{x}\right)|\cdot B_{i}^{\ell }\left(\text{x}\right)\cdot B_{j}^{\ell }\left(\text{x}\right)\right)/C_{ma\text{x}}$$

(5) $$B_{i}^{\ell }\left(\text{x}\right)=\left\{\begin{array}{cc} 0 & if\sum_{\ell_{0}=1}^{\ell -1}V_{\ell_{0}}^{i}\left(\text{x}\right)>0\\ 1 & otherwise \end{array}\right.$$

$I_{i}^{d}\left(\text{x}\right)$ represents the value of pixel x on the warped image of camera *i* in depth *d*. $B_{i}^{\ell }\left(\text{x}\right)$ is a binary map of camera *i* to mask fully-visible pixels of previous layers. $C_{ma\text{x}}$ is the maximum gray intensity, and for a eight-bit camera, its value is 255. $V_{\ell_{0}}^{i}$ is the visibility layer $\ell_{0}$ of camera *i* and can be obtained easily from $V_{\ell_{0}}$ of the reference camera. If $B_{i}^{\ell }\left(\text{x}\right)=0$, x is occupied by previous layers, otherwise $B_{i}^{\ell }\left(\text{x}\right)=1$.

A good energy function should reach a good solution when the energy is low. In order to achieve this, we design the data term of the visibility optimization model as Equation (3), which is introduced to classify all of the pixels as visible or invisible. When min(MCD) is small, or the data term is small, the probability that the point is occluded is low; thus, the cost of assigning it as a visible point is low. In addition, according to the definition of MCD, even if one of the camera views is occluded, the min(MCD) appears to be a large value, and the cost of assigning this point as a visible point is high by Equation (3). Thus, for visibility labeling, it is straightforward to see that our data term should achieve its minimum when it is correctly assigned and achieve a large value for an occluded point, which is the perfect data term that we want.

Smoothness term: The smoothness term $E_{s}\left(V_{\ell }\right)$ at layer *ℓ* is a prior regularizer that encourages overall labeling to be smooth. The prior is that two neighboring pixels have a higher probability to belong to the same object and should be both visible or occluded in the reference camera at the same time. Here, we adopt the standard four-connected neighborhood system and penalize the fact if labels of two neighboring pixels are different:

(6) Es(Vℓ)=∑p∈Irefq∈𝒩pSp,q(Vℓ(p),Vℓ(q))

(7) $$S_{p,q}\left(V_{\ell }\left(p\right),V_{\ell }\left(q\right)\right)=\min(\tau_{v},\beta \left(p,q\right)\cdot |V_{\ell }\left(p\right)-V_{\ell }\left(q\right)\left|\right)$$

(8) $$\beta \left(p,q\right)=h(|\min_{d\in \text{𝒟}}\left({MCD}^{d}\left(p\right)\right)-\min_{d\in \text{𝒟}}\left({MCD}^{d}\left(q\right)\right)|)$$

where $\tau_{v}$ and β(p,q) denote the maximum and weight of the smoothness term, respectively. *h* is a decreasing weighting function that takes into account the MCD measure similarity between neighboring pixels. The more similar the MCD measure is, the weight will be higher, and the smoothness constraint between pixels will be stronger.

Because every two neighboring pixels have a higher probability of belonging to the same object and should be both visible or occluded in the reference camera at the same time, so the MCD measure should be similar between neighboring pixels, which means that the smoothness term will be big between neighboring pixels. In our algorithm, in order to represent the decreasing relationship between the MCD measure and the smoothness term, we simply use the inverse proportional function h(x)=k/x as h(.) in our experiments. It is worth noting that other functions that can represent the decreasing relationship should also work well. With the above data term and smoothness term, our energy function can be minimized via graph cuts [22].

## 4. Multiple-Layer Depth Optimization

As $V_{\ell }$ is obtained by the visibility optimization in Section 3, we could estimate the optimal depth of these fully-visible pixels by multiple depth label optimization. Different from the visibility optimization, which applies the graph cuts to get all visibility layers, our multiple-layer depth map optimization model applies the graph cuts to get the optimal focus depth of the fully-visible pixels in each visibility layer. In this model, we also need to find a labeling function: $D_{\ell }:{\text{Ω}}_{\ell }\to \text{𝒟}$, where ${\text{Ω}}_{\ell }=\left\{\text{x}:|V_{\ell }\left(\text{x}\right)=1\right\}$ represents the set of all fully-visible pixels on the reference camera and 𝒟 is the depth range of the scene. In order to get this labeling function, we use the energy minimization framework to do this work:

(9) $$E\left(D_{\ell }\right)=E_{d}\left(D_{\ell }\right)+E_{s}\left(D_{\ell }\right)$$

Data term: The data term should reflect defocusing and reaches the global minimal at the optimal focusing depth. Just as we have analyzed in Section 3, the MCD metric outperforms other focus measures; thus, we adopt the MCD measure in the synthetic aperture images as the cost function:

(10) $$E_{d}\left(D_{\ell }\right)=\sum_{\text{x}\in {\text{Ω}}_{\ell }}{MCD}^{D_{\ell }\left(\text{x}\right)}\left(\text{x}\right)$$

Smoothness term: The smoothness term $E_{s}\left(D_{\ell }\right)$ of layer *ℓ* is a regularizer that encourages the overall depth labeling to be smooth. Similar to Section 3, we adopt the standard four-connected neighborhood system and penalize the fact that the depth labels of two neighboring pixels are different.

(11) Es(Dℓ)=∑p∈Ωℓq∈𝒩pSp,q(Dℓ(p),Dℓ(q))

(12) $$S_{p,q}\left(D_{\ell }\left(p\right),D_{\ell }\left(q\right)\right)=\min(\tau_{v},\beta \left(p,q\right)\cdot |D_{\ell }\left(p\right)-D_{\ell }\left(q\right)\left|\right)$$

where $\tau_{v}$ and β(p,q) are as defined in Section 3. Again, the problem of depth label optimization is solved via graph cuts [22].

Because we already get all synthetic images of different depths of focus, so after obtaining $V_{\ell }$ and $D_{\ell }$, we can get all pixel’s optimal imaging results and each layer’s color information. Combing with the visible information in each visibility layer and removing the visibility layer that contains the occluders, we can get the all-in-focus imaging result just by simply combining the remaining layers.

The above process also can be represented by:

(13) $$I_{syn}^{\ell }\left(x\right)=\frac{1}{\sum_{k=1}^{N}B_{k}^{\ell }\left(x\right)}\sum_{k=1}^{N}B_{k}^{\ell }\left(x\right)\cdot I_{k}^{D_{\ell }\left(x\right)}\left(x\right)$$

where *x* is one pixel in the reference camera, $I_{syn}^{\ell }\left(x\right)$ is the synthetic value of pixel *x* in $V_{\ell }$, $D_{\ell }\left(x\right)$ denotes the optimal depth of pixel *x* in $V_{\ell }$ and $I_{k}^{D_{\ell }\left(x\right)}\left(\text{x}\right)$ represents the value of pixel x on the warped image of camera *k* at depth $D_{\ell }\left(x\right)$. *N* represents the number of camera views. Then, given a depth $d_{f}$, we could generate the all-in-focus synthetic aperture image by combining $I_{syn}^{\ell }\left(x\right)$ with $D_{\ell }\left(x\right)>d_{f}$.

## 5. Experimental Results

We have compared the performance of our method with the synthetic aperture imaging methods of Vaish *et al.* [11] and Pei *et al.* [6] on four datasets, including the CD case behind plants from Stanford, the crowd surveillance scene from UCSD and two datasets captured by ourselves. In addition, to illustrate that our method can be successfully applied when there are multiple visibility layers, we have captured another dataset where there are multiple occluders.

To avoid explicit imaging for all of the objects that are far away in the scene, we limit our search to a range of depths around the objects that are our concern. For the CD case behind plants from Stanford and our own dataset, the accuracy of each method is compared to the ground truth separately. More implementation details are given below.

### 5.1. Experiment 1: CD Case behind Plants

This dataset contains 105 views on a 21 × 5 grid (synthetic aperture size 60 cm by 10 cm), and the image resolution is 650 × 515. The scene contains some plants occluding two CD cases. Our goal is to estimate the depth for all of the objects in the scene and to image the scene behind the plants.

**Figure 6:**
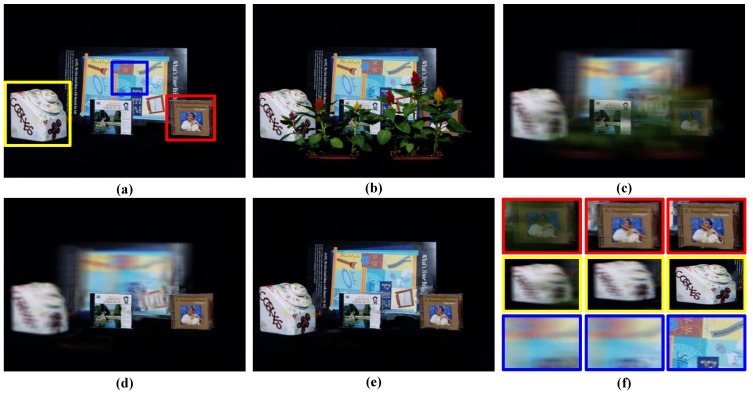
Comparison results of different methods on the CD case behind plants from Stanford. (**a**) Ground Truth; (**b**) Reference camera view; (**c**) Result of Vaish *et al.* [11]; (**d**) Result of Pei *et al.* [6]; (**e**) All-in-focus image by our method; (**f**) Comparison results, Column 1 is Vaish’s result, Column 2 is Pei’s result and Column 3 is our method’s result.

Figure 6 shows the comparison results of Vaish *et al.* [11], Pei *et al.* [6] and our method. From Figure 6c–e, we can see that all three methods could see through the plants and get the imaging result of the occluded CD cases. Additionally, for the certain focus plane, which contains the CD cases, Pei *et al.* [6] is clearer than Vaish *et al.* [11]. However, the objects off this focus plane are still blurred, including the CD on the right, which is just near the focus plane. The all-in-focus image in Figure 6e shows much better clarity of our method and is closer to the ground truth in Figure 6a.

Figure 6f gives the comparison of the imaging result for several local regions. It can be seen that our method can give the all-in-focus image for all three objects of different depths, while the methods of Vaish *et al.* [11] and Pei *et al.* [6] can only focus on a given depth plane.

Besides, we use the peak signal-to-noise ratio (PSNR) assessment to compare these methods quantitatively (see Table 1). The calculation of PSNR is given in Equations (14) and (15).

(14) $$PSNR=10\log_{10}\left(I_{\max}^{2}/MSE\right)$$

(15) $$MSE=\frac{1}{w\cdot h}\sum_{\text{x}\in \text{𝒳}}{\left(I\left(\text{x}\right)-I^{\prime }\left(\text{x}\right)\right)}^{2}$$

where *w* and *h* denote the image width and height, 𝒳 is the image region, I(x) is the pixel intensity value at x in the ground truth image and $I^{\prime }$ denotes the image to be assessed. $I_{\max}=255$ is the maximal intensity value.

**Table 1:** PSNR of CD cases by different methods.

METHODS	MSE	PSNR
**Vanish’s Method** [11]	1001.9	18.1225
**Pei’s Method** [6]	531.2978	20.8774
**Our Method**	50.3735	31.1088

As we all know, for different methods, the more clarity of imaging that results, the bigger the PSNR value that will be achieved. In this experiment, the PSNR that our all-in-focus synthetic aperture image achieves is 31.1088, which is much higher than 20.8774 of Pei *et al.* [6] and 18.1225 of Vanish *et al.* [11]. Therefore, compared to other methods, the all-in-focus imaging result by our method is clearer and closer to the ground truth.

### 5.2. Experiment 2: Crowd Surveillance Scene

The “Crowd” dataset is captured by UCSD with eight synchronous views on an 8 × 1 grid. There are 276 frames, and the image resolution is 640 × 480. The scene contains five people moving in the scene, and they are frequently occluded by each other. Our goal is to see through the occluder in the front and to image for all others continuously.

**Figure 7:**
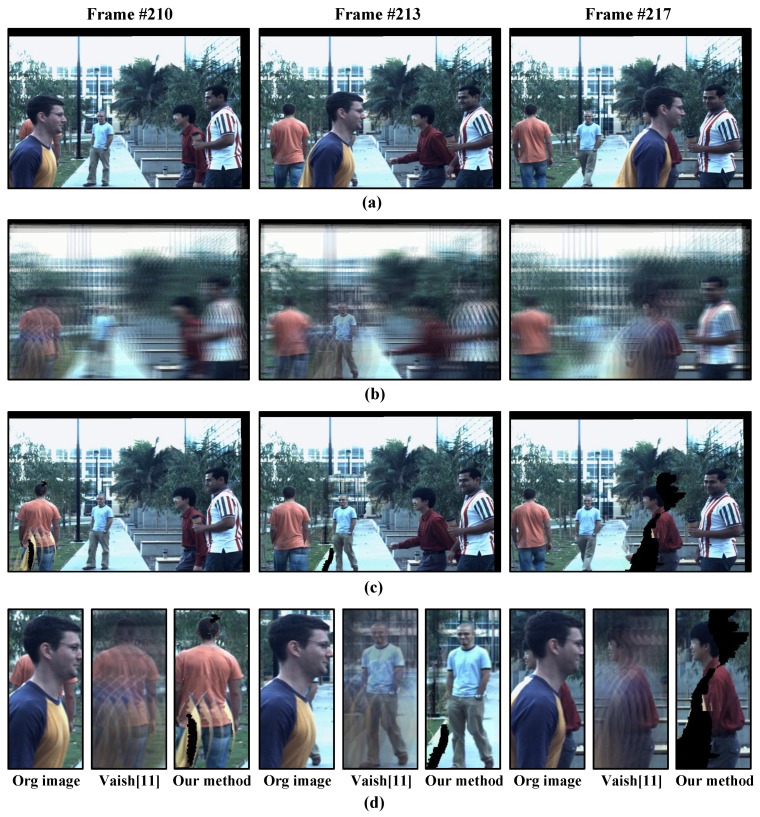
Comparison results on crowd surveillance data from UCSD light field data. (**a**) Original sequences of reference camera view; (**b**) Occluded object imaging result of Vaish *et al.* [11]; (**c**) Occluded object imaging result by our method; (**d**) Comparison of local region.

Figure 7 shows the comparison result of our method and Vaish *et al.* [11]. In Frame 210, the man in saffron cloth is occluded. In Vaish’s result (Figure 7b), the occluded man is blurred by shadows from the occluder. In addition, people out of the focus plane are all blurred. In contrast, our approach could see through occlusion and achieve a clear all-in-focus image (Figure 7c). The details of the local region results are shown in Figure 7d. Our method also shows better performance in Frames 213 and 217 than Vaish’s method.

The success of our work comes from the idea that for every synthetic aperture imaging result on each frame, the scene can be regarded as static, and there are no moving objects. This is quite reasonable, as no object would make obvious movements considering the high frequency of the camera. Figure 7 shows the result of several subsequent frames.

One limitation of our approach is that a scene point needs to be visible at least in two camera views; otherwise, a black hole will appear in the all-in-focus image (Figure 7c).

### 5.3. Experiment 3: Complex Outdoor Scene

**Figure 8:**
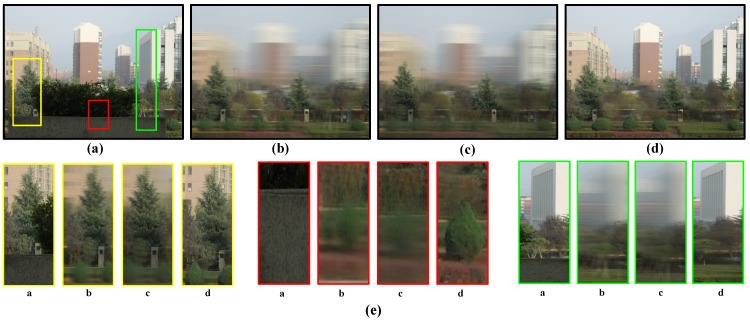
Comparison results of different methods in the challenging outdoor scene. (**a**) Reference view; (**b**) Result of Vaish’s method; (**c**) Result of Pei’s method; (**d**) Result of our method; (**e**) Zoom images.

To further test our method on severe occlusion cases, we have done another experiment with a complex outdoor scene. As shown in Figure 8, the street, trees and distant buildings are all occluded by nearby flowers. Our aim is to see behind the scene through the occlusion of the flowers in front. In this experiment, we adopt the ACTS system developed by Zhang *et al.* [29] for camera pose estimation. Comparison results of Vaish’s method [11], Pei’s method [6] and our method are shown in Figure 8b–d. As Vaish’s method only focuses on a given depth plane and cannot eliminate front occluders completely, it cannot provide an all-in-focus image of the scene behind. Additionally, although Pei’s method can remove some foreground occluders through foreground occluder segmentation and get a more clear result for the target, the targets’ out-of-focus plane is still very blurred, for example the building shown in Figure 8e. In comparison, our method could provide a depth-free view point and all-in-focus image for any given depth range. For instance, Figure 8d shows the all-in-focus image of the scene behind the flowers. Please note that although the depth change and occlusion in this scene are extremely complex, our method accurately gives the desired all-in-focus result.

### 5.4. Experiment 4: Seeing through Multiple Occluded Objects

**Figure 9:**
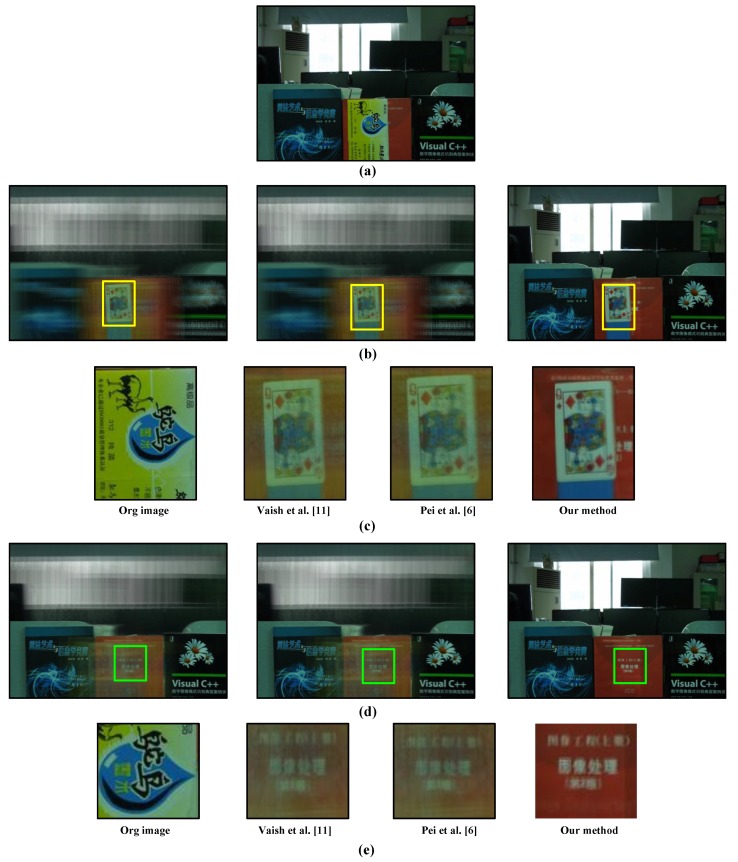
Comparison results of synthetic aperture imaging through multiple objects. (**a**) Original reference image; (**b**) Imaging result through the front object by Vaish, Pei and our method; (**c**) Comparison results through the first occluder; (**d**) Imaging result through the second object by Vaish, Pei and our method; (**e**) Comparison results through the second occluder.

Our method can be applied to a scene where there are multiple occluded objects. Due to visibility propagation between different layers, we can remove multiple occluders, focus on the occluded object and obtain an all-in-focus image of the occluded scene. Figure 9 shows the result of our synthetic aperture imaging method when there are multiple occluders. Figure 9a is the input image of the reference camera; it can be seen that the red book is occluded by the playing card, which is further occluded by the yellow box in front. The standard synthetic aperture imaging result of the playing card and red book is shown in Figure 9b,e, respectively. It can be seen that due to severe occlusion, Vaish’s method [11] can only get a blurred image of the occluded object. The state-of-the-art synthetic aperture imaging result of the playing card and red book is shown in Figure 9c,f, respectively. It can be seen that in the case of severe occlusion, Pei’s method [6] can only get a blurred image of the occluded object due to the inaccuracy of the estimated foreground label. In comparison, our method can remove front occluders completely and provide an all-in-focus image of the scene behind the yellow box (Figure 9d) and even the playing card (Figure 9g).

## 6. Conclusions and Future Work

In this paper, we have presented a novel synthetic aperture imaging approach for creating all-in-focus images through occlusion. Different from existing synthetic aperture imaging algorithms, we have segmented the scene into multiple visibility layers and apply an optimization framework to propagate the visibility information between multiple layers to produce an all-in-focus image, even under occlusion.

We believe this approach is useful in challenging applications, like surveillance of occluded people in crowded areas, where seeing the people’s appearance may be of primary interest, or reconstructing hidden objects through severe occlusion, or even rendering a depth-free viewpoint image. In the future, we would like to design more robust cost functions for the focus depth estimation and to extend our work to unstructured light field imaging through occlusion with handheld mobile phones.
